# Patients’ Expectations and Satisfaction with the Patient–Doctor Relationship in Hidradenitis Suppurativa

**DOI:** 10.3390/healthcare11243139

**Published:** 2023-12-11

**Authors:** Julia Ewa Rymaszewska, Maciej Karczewski, Piotr K. Krajewski, Łukasz Matusiak, Joanna Maj, Jacek C. Szepietowski

**Affiliations:** 1 Department of Dermatology, Allergology and Venereology, Wrocław Medical University, T. Chałubińskiego Str. 1, 50-368 Wrocław, Polandpiotr.krajewski@umw.edu.pl (P.K.K.); luke71@interia.pl (Ł.M.); joanna.maj@umw.edu.pl (J.M.); 2 Department of Applied Mathematics, Wrocław University of Environmental and Life Sciences, Grunwaldzka Str. 53, 50-357 Wrocław, Poland; maciej.karczewski@upwr.edu.pl

**Keywords:** hidradenitis suppurativa, acne inversa, patient–doctor relationship, satisfaction with life, psychopathological symptoms

## Abstract

Introduction: Hidradenitis suppurativa (HS) is a chronic inflammatory dermatosis with a vast psychosocial burden. We analyzed the actual and ideal patient–doctor relationship and patients’ satisfaction with the patient–doctor relationship in relation to their satisfaction with life (SWL), HS-related quality of life, and psychopathological symptoms. Methods: 105 HS patients (53% females; mean age 37.64 ± 14.01 years) were enrolled. Severity of the disease was measured using Hurley staging and the International HS Score System (IHS4). Instruments utilized: Patient Expectation Test; Satisfaction with Life Scale; HS Quality of Life; Patient Health Questionnaire-9; Generalized Anxiety Disorder-7; General Health Questionnaire. Results: Patients with Hurley I and mild IHS4 had the lowest satisfaction with the patient–doctor relationship. There were significant correlations between the actual patient–doctor relationship and the patients’ SWL (r = 0.30; *p* = 0.002), depressive (r = −0.36; *p* < 0.01), anxiety (r = 0.37; *p* < 0.01) and psychopathological symptoms (r = −0.47; *p* < 0.0001) and between the satisfaction with the patient–doctor relationship and their SWL (r = −0.32; *p* = 0.00098). Multiple regression analysis revealed a significant influence of the following factors: Hurley II + III, psychopathological symptoms, and severe anxiety about the actual patient–doctor relationship and the satisfaction with the patient–doctor relationship. Conclusions: Assessment of relations between patients and doctors is related to the patients’ mental health and SWL. The usage of the Patient Expectation Test in clinical practice can improve the patient–doctor relationship and the general quality of care for and compliance by HS patients.

## 1. Introduction 

Hidradenitis suppurativa (HS), also known as acne inversa, is a chronic, recurrent, inflammatory dermatosis of multifactorial, but not fully understood, etiology [1]. The disease is characterized by the occurrence of deeply located inflammatory lesions (nodules, abscesses, and fistulas), often affecting the anogenital area, buttocks, armpits, and groin [1]. The treatment of HS often proves to be a great therapeutic challenge [2]. Patients suffering from HS often report a dissatisfaction related to the treatment outcomes [3]. Due to the clinical picture, associated pain, and the characteristic location of lesions, HS is usually associated with significant levels of stigma as well as depression and anxiety in affected patients [4,5]. It has a negative impact on patients’ quality of life and day-to-day functioning [6]. Furthermore, there is a significant influence by accompanying symptoms of HS, such as pain in over half and pruritus in over 80% of the patients on the sleep quality of the individuals affected by the disease [7]. Moreover, recent literature states that HS patients present indirect self-destructive behaviors such as: transgression and risk, poor health maintenance, personal and social neglect, a lack of planfulness, and helplessness and passiveness in the face of problems or difficulties [8]. Additionally, studies found that the prevalence of a psychological comorbidity such as alexithymia was more frequent among patients with HS compared with healthy controls [9]. Patients still experience a serious delay in the diagnosis, even up to several years [10]. An average of 7 years usually passes between the start of the primary symptoms and the confirmation of the diagnosis [11]. According to studies, people with HS experience a more prolonged diagnostic delay than those with psoriasis [6,12]. This delay might be the result of the patient delaying a visit to a medical professional, the physician making an incorrect diagnosis, or, simply, difficult access to a dermatology specialist [6,13]. Another study found that nearly 80% of the HS patients with a moderately late diagnosis and nearly 90% of individuals with a late diagnosis have been misdiagnosed compared with 46.5% of patients with an early HS diagnosis. Additionally, there is a positive correlation between the number of misdiagnoses and the length of the diagnosis delay [14]. Recent studies suggest a major role of ultrasound in early diagnosis, especially of the non-clinically evident HS lesions [15,16].

Our recent study suggested that a reasonable number of HS patients suffered from mental disorders, namely depression and anxiety [17]. Patient satisfaction and dissatisfaction are the key markers of the quality of a medical consultation. It has been demonstrated that patient satisfaction is influenced by the diagnosis as well as the doctor’s capacity to explain the potential cause of the disease, offer information on how long the symptoms are likely to continue, and most importantly, whether the physician demonstrates empathy [18]. In clinical practice, the connection between the patient and the medical practitioner is a crucial topic [19]. Since psychological issues are frequently linked to skin illnesses, they are crucial components of a thorough clinical examination of the disease [18].

Therefore, it is of great importance to not only assess the raw clinical picture and be cautious while assessing patients’ symptoms and skin lesions but also to determine the patient expectations toward their physician, as well as to create a trusting patient–doctor relationship. Nevertheless, the literature on the abovementioned topics in relation to HS is noticeably limited. 

Hence, the objective of the present study is to thoroughly analyze patients’ expectations and the actual and ideal patient–doctor relationship in relation to their satisfaction with life, HS-related quality of life, as well as psychopathological symptoms. 

## 2. Materials and Methods

### 2.1. Participants and Study Design

Our cross-sectional study enlisted 105 consecutive patients suffering from HS from two Polish centers. The inclusion criteria were patients who, after receiving a detailed information about the study, agreed to take part in it as well as patients with a diagnosis of HS. We excluded patients under the age of 18. Our study included 56 (53.3%) females and 49 (46.7%) males. The mean age was 38.32 ± 13.30 years (Table 1). This study received approval from the Wroclaw Medical University Bioethics Committee (KB-901/2022). The data was collected from two different regions of Poland (south-west and south-east Poland) between September 2020 and September 2021. The first part of our questionnaire consisted of basic demographic data, such as sex and age. The second part included clinical factors regarding HS; specifically, the number of hospitalizations, and the duration of the disease (mean: 9.53 ± 8.17 years) (Table 1). The questionnaire also collected data on the severity of HS using a given set of questionnaires in validated Polish language versions.

### 2.2. Assessments 

#### 2.2.1. HS Severity

In order to determine the severity of HS, two methods were used: the Hurley staging system [20] and the International Hidradenitis Suppurativa Severity Score System (IHS4) [21]. 

The Hurley staging system divides patients into three groups based on the presence and extent of lesions, scarring, and sinus tracts. Hurley stage I involves single or multiple inflammatory nodules or abscesses without scarring and sinus tracts. Hurley stage II involves recurrent abscesses or nodules with sinus tract formation and scarring (frequently with several individual lesions present), while Hurley stage III involves widespread involvement with multiple intertwined sinus tracts, abscesses, and scarring [20]. 

On the other hand, the IHS4 is a validated tool that assesses the clinical severity of HS by counting the number of nodules, abscesses, and draining tunnels, with points assigned to each based on the following formula: (number of nodules × 1) + (number of abscesses × 2) + (number of draining tunnels × 4) [21]. The severity of HS is then categorized into mild, moderate, and severe based on cut-off points: up to 3 points for mild HS, 4–10 points for moderate HS, and above 10 points for severe HS [20,21].

#### 2.2.2. Satisfaction with the Patient–Doctor Relationship 

The Patient Expectation Test (Goldzweig test) [22] was used in the study. It is a tool containing eight items describing the actual and ideal relationship between a doctor and a patient in terms of emotional support, providing information about the disease, and treatment, both to the patient and his family. The questionnaire is intended for self-completion by patients. With regard to individual issues, the respondent, on the basis of a 4-point scale, answers to what extent he/she agrees with the given statement (1 point—complete disagreement; 4 points—agreement to a very large extent). The test analyzes three dimensions: (1) assessment of the real, actual course of the relationship between the doctor and the patient (min. score = 8 points; max. score = 32 points); (2) assessment of the expected course of the relationship—called “ideal relationship” (min. score = 8 points; max. score = 32 points); (3) assessment of the satisfaction with the course of contact with a doctor in the scope of the analyzed issues. Satisfaction with the patient–doctor relationship is defined as the difference between the expectation of an “ideal relationship” and the assessment of the actual situation. One can talk about high satisfaction with the relationship when the patient’s expectation corresponds to the situation currently experienced.

#### 2.2.3. Satisfaction with Life

Satisfaction with life (SWL) was assessed with the Satisfaction with Life Scale (SWLS) [23]. SWLS is a 5-item scale where a patient evaluates how much each of the item corresponds to his or her life so far, rated on a 7-point scale: from 1 point denoting “I completely disagree” to 7 points denoting “I completely agree”. The overall mark is the sum of all scores. The range of results ranges from 5 to 35 points: the higher the score, the greater the sense of satisfaction with life. The data were transformed to a sten scale to ascertain the sense of SWL. Results in the 1- to 4-point range are given as low, results in the 5- to 6-point range are presented as average, and results in the 7- to 10-point range are displayed as a high SWL [23].

#### 2.2.4. Quality of Life Related to HS

The Hidradenitis Suppurativa Quality of Life Scale, HiSQoL [24], is a scale consisting of 17 items designed to assess the patients’ quality of life and their symptoms and emotions related to the disease over the last 7 days. Respondents utilize a 5-point scale to rate their experiences that consolidates responses as: “extremely”, “very much”, “moderately”, “slightly” and “not at all” with 4, 3, 2, 1, and 0 points respectively [25]. The questionnaire also includes additional items like “unable to do, due to my HS” (score: 4 points) and/or “I do not normally do this, HS did not influence” (score: 0 points). The HiSQoL questionnaire was further divided into three subscales: activities–adaptations, psycho-social, and symptoms [26].

#### 2.2.5. Psychopathological Symptoms

The mental status of the participants over the past two weeks was evaluated using two different questionnaires—the Patient Health Questionnaire-9 (PHQ-9) [27] and the Generalized Anxiety Disorder-7 (GAD-7) [28]. Each item in both scales can be rated on a scale of 0 to 3 points (with 0 indicating “not at all”, 1 indicating “several days”, 2 designating “more than half the days”, and 3 indicating “nearly every day”). The PHQ-9 scale consists of nine items that assess the following: feeling sad, depressed, or hopeless; sleep disturbance; lack of energy; appetite changes; problems with focusing on certain tasks as well as thoughts about hurting oneself or death. The total score of PHQ-9 ranges between 0 and 27 points, with cut-off points of 5 (mild), 10 (moderate), 15 (moderately severe), and 20 points (severe depression). The GAD-7 scale has seven questions that evaluate the sense of anxiety, tension, nervousness, the ability to control these feelings, the ease with which they appear, and difficulty relaxing. The total score of GAD-7 ranges between 0 and 21 points, with cut-off points of 5 (mild), 10 (moderate), and 15 points (severe anxiety) [22,23]. General Health Questionnaire-28 (GHQ-28) [29] is a 28-item scale used to screen for minor psychiatric and non-psychotic disorders. It is divided into four subscales: somatic symptoms, anxiety/insomnia, social dysfunction, and severe depression. Each item can be scored from 0 to 3 points for each response, with the total possible score ranging from 0 to 84 points. While utilizing this method, a total score of 23 is the threshold for the presence of distress. Alternatively, GHQ-28 can be assessed with a binary method, where score 0 is assigned to “not at all” and “no more than usual” and score 1 to “rather more than usual” and “much more than usual”. While utilizing this approach, any score above 4 points indicates the presence of distress [29].

### 2.3. Statistical Analysis

Differences between the groups when analyzing the patient–doctor relationship, levels of SWL, clinical severity of HS, and the HiSQoL were assessed with the Kruskal–Wallis test. The relationships between the variables were assessed by the Spearman correlation. Post hoc analysis was performed to establish if the different variables influence other variables independently. We used the Spearman correlation coefficient to analyze how the patient–doctor relationship correlates with the SWLS, HiSQoL, PHQ-9, GAD-7, or GHQ-28 questionnaires. The independent effect of variables on the patient–doctor relationship was performed utilizing multiple regression analysis. Each model was additionally adjusted for sex, duration of disease, and HiSQoL. Analysis was performed in R for Windows (version 4.3.1, Vienna, Austria) [30]. Graphics were made using the “ggstatsplot” package [31]. All tests with *p* < 0.05 were considered statistically significant. 

## 3. Results

### 3.1. Clinical HS Severity

According to Hurley staging, the majority of our patients (69 subjects; 65.7%) presented with Hurley stage II, 26 patients (24.8%) were diagnosed with Hurley stage I, and the remaining 10 (9.5%) with Hurley stage III. In relation to cut-off points of the IHS4, 25 patients (24%) suffered from mild HS, 38 (36%) from moderate HS, and 42 subjects (40%) had severe disease. The mean duration of HS was 9.53 ± 8.17 years (Table 1). 

### 3.2. Satisfaction with the Patient–Doctor Relationship 

Based on the *Patient Expectation Test scores* of our group, the mean satisfaction with the patient–doctor relationship among men was numerically higher (1.27 ± 4.87 points) than among women (3 ± 5.94 points); however, the difference did not reach statistical significance. Additionally, we have not established statistically significant differences in either sex regarding the actual and ideal patient–doctor relationship (Table 2). 

### 3.3. Satisfaction with Patient–Doctor Relationship and Clinical HS Severity

Regardless of the severity of the disease, assessed by Hurley staging and the IHS4, the ideal and actual relationships as well as the satisfaction with the patient–doctor relationship were similar. Moreover, there were no correlations between disease severity and satisfaction with the patient–doctor relationship or with actual and ideal relationships. 

### 3.4. Patient–Doctor Relationship and Satisfaction with Life

**Figure 1:**
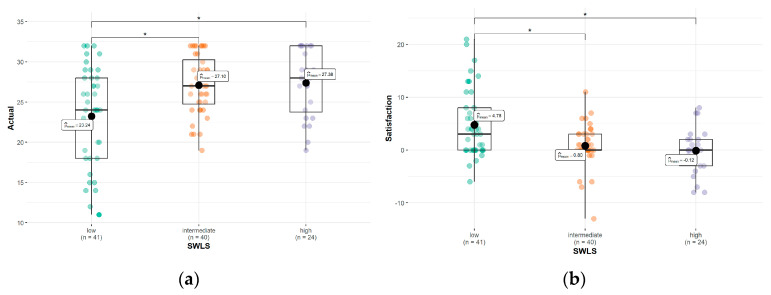
Differences between the actual (**a**) and satisfaction with (**b**) the patient–doctor relationship among groups with low, intermediate, and high SWL. *—statistically significant difference.

There was a positive significant correlation (r = 0.30; *p* = 0.002) between the actual patient–doctor relationship and the SWL. Additionally, we documented a statistically significant difference in the actual patient–doctor relationship between patients with high and low SWL (*p* = 0.018) as well as between individuals with low and intermediate SWL (*p* = 0.018) (Figure 1a). 

Moreover, a negative significant correlation (r = −0.32; *p* < 0.001) between satisfaction with the patient–doctor relationship and SWL was found. Taking into consideration the SWLS cut-off points, we found statistically significant differences in the satisfaction with the patient–doctor relationship between patients with high and low SWL (*p* = 0.011) as well as between the patients with low and intermediate SWL (*p* = 0.019) (Table 3; Figure 1b). 

### 3.5. Patient–Doctor Relationship and Quality of Life (HiSQoL)

The actual patient–doctor relationship scores significantly correlated weakly and negatively (r = −0.23; *p* = 0.018) with the HS-related quality of life (HiSQoL). No correlations were found for the quality of life and satisfaction with the patient–doctor relationship as well as between the quality of life and the ideal patient–doctor relationship.

### 3.6. Patient–Doctor Relationship and PHQ-9

We established statistically significant differences in the *Patient Expectation Test* data in HS patients with different levels of PHQ-9 severity (Table 4). A positive significant correlation (r = 0.33; *p* < 0.01) between the satisfaction with the patient–doctor relationship score and depressive symptoms measured by the PHQ-9 scale was found.

**Figure 2:**
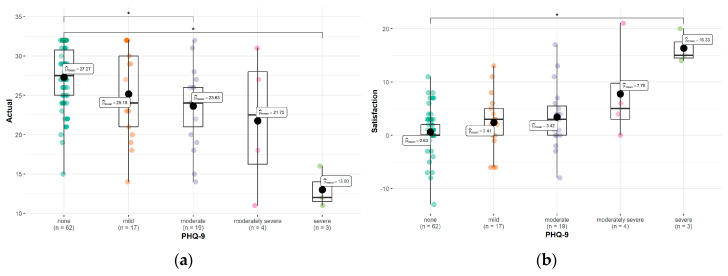
Differences between the actual (**a**) and satisfaction with (**b**) the patient–doctor relationship among groups with no, mild, moderate, moderately severe, and severe depressive symptoms. *—statistically significant difference.

Taking into consideration the PHQ-9 cut-off points, we found statistically significant differences in the actual patient–doctor relationship scores between the patients with moderate depression and no depression (*p* = 0.018) as well as between the patients with severe and no depression (*p* = 0.036) (Figure 2a). Moreover, we saw a statistically significant difference in the satisfaction with the patient–doctor relationship scores between the patients with severe and no depression (*p* = 0.029) (Figure 2b). Furthermore, a negative significant correlation (r = −0.36; *p* < 0.01) between the actual patient–doctor relationship score and depressive symptoms measured by the PHQ-9 scale was found.

### 3.7. Patient–Doctor Relationship and GAD-7

**Figure 3:**
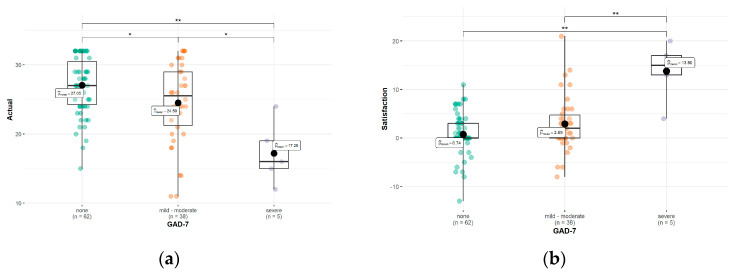
Differences between the actual (**a**) and satisfaction with (**b**) the patient–doctor relationship among groups with no, mild, moderate, and severe anxiety symptoms. *, **—statistically significant difference.

The satisfaction with the patient–doctor relationship and actual relationships differ significantly between groups of HS patients presenting various severities of anxiety (Table 5). Taking into consideration the GAD-7 cut-off points, we saw a statistically significant difference in the actual patient–doctor relationship scores between the patients with mild-moderate and severe anxiety (*p* = 0.035) as well as between the patients with severe and no anxiety (*p* = 0.003) (Figure 3a). Additionally, we showed statistically significant differences in the scores of satisfaction with the patient–doctor relationship between the patients with severe and mild–moderate anxiety (*p* = 0.006) as well as between the patients with severe and no anxiety (*p* = 0.001) (Figure 3b). There was a negative significant correlation (r = 0.37; *p* < 0.01) between the actual patient–doctor relationship scores and anxiety symptoms measured by the GAD-7 scale. Moreover, the assessment of patients’ satisfaction with the patient–doctor relationship correlated positively and significantly (r = 0.33; *p* = 0.0006) with the anxiety symptoms. 

### 3.8. Patient–Doctor Relationship and GHQ-28

We identified a negative, moderate, significant correlation (r = −0.47; *p* < 0.0001) between the actual patient–doctor relationship score and the presence of psychopathological symptoms measured by GHQ-28. We also found significant negative correlations between the actual patient–doctor relationship score and the following domains of the GHQ-28 questionnaire: somatic symptoms (r = −0.50; *p* < 0.0001), anxiety and insomnia (r = −0.40; *p* < 0.0001), social dysfunction (r = −0.37; *p* < 0.0001), and severe depression (r = −0.32; *p* < 0.0001). A positive, moderate, significant correlation (r = 0.43; *p* < 0.0001) between the satisfaction with the patient–doctor relationship score and the presence of psychopathological symptoms was established. Additionally, the subsequent significant positive correlations between the satisfaction with the patient–doctor relationship score and the following GHQ-28 questionnaire domains were shown: somatic symptoms (r = 0.46; *p* < 0.0001), anxiety and insomnia (r = 0.37; *p* < 0.0001), social dysfunction (r = 0.35; *p* < 0.0001), and severe depression (r = 0.30; *p* = 0.0020).

### 3.9. Multiple Regression Analysis 

The multiple regression analysis (Table 6) showed a significant influence of the following factors: Hurley grades II + III, total GHQ-28 and no anxiety (GAD-7) as well as severe anxiety (GAD-7) on the actual patient–doctor relationship (*p*-values: 0.010, 0.001, 0.365, 0.034, respectively). Additionally, the multiple regression analysis revealed the significant influence of Hurley grades II + III, total GHQ-28 and no anxiety (GAD-7) as well as severe anxiety (GAD-7) on the satisfaction with the patient–doctor relationship (*p*-values: 0.003, 0.001, 0.344, 0.002, respectively) (Table 6). A regression model was also created for the ideal patient–doctor relationship; however, the results were insignificant.

## 4. Discussion

This study’s objective was to define and assess the patient–doctor relationship among HS patients as well as their satisfaction with the relationship. Despite the importance of the patient–doctor relationship, up until now, this factor seems to have attracted little attention in the area of HS in the scientific literature. Different modalities such as depression, anxiety, and SWL can influence patients’ answer to the self-assessed questionnaires. To the best of our knowledge, this is the first publication which has utilized the Patient Expectation Test among a cohort of HS patients. However, this tool has been utilized in a group patients with cancer [22]. Our findings illustrate the complex relationships between patients with HS and their doctors. An interesting outcome was that the patients with low SWL rate their actual patient–doctor relationship lower than the patients with intermediate and high SWL. Furthermore, a notable and intriguing result was that the level of satisfaction of the patient–doctor relationship of patients with high SWL was significantly higher than the patients anticipated. A somewhat similar result on a different cohort of patients with cancer was established in a study by Goldzweig et al. [32] When asked about the ideal patient–doctor relationship, not all of the patients expected or desired the highest level of support from their oncologists [32]. Moreover, we determined that the individuals with a lower quality of life due to HS rated their actual patient–doctor relationship higher. From a psychological point of view, we can hypothesize that individuals who have a lower quality of life living with a chronic severe disease rate their actual relationship higher because they are hoping to be well taken care of by the physician. This is supported by the results achieved in a study by Renzi et al. [33] In their paper, when the symptom-related quality of life decreased, the patient satisfaction with care increased [33]. Moreover, our analysis showed that the higher the anxiety level, the worse the assessment of the patient–doctor relationship. Conversely, patients with no anxiety or mild anxiety levels had the highest satisfaction with the patient–doctor relationship. Additionally, the actual patient–doctor relationship was the best among patients with no anxiety and the lowest among patients with severe anxiety. Again, from a psychological point of view, it is understandable that a high level of anxiety creates doubts and further possible restlessness, which, consequently, leads to a lower evaluation of satisfaction with the patient–doctor relationship. A study conducted by Linder et al. [34] on a group of 300 patients with psoriasis, during recorded discussions in focus groups, established that 28.3% of them presented with anxiety emotions. This is an important discrepancy, since in our group of HS patients, 41.0% had anxiety symptoms [17]. But also, we have to take into consideration that their methodology was based on qualitative, descriptive methods of analyzing patients’ emotions. Among those individuals, the focus groups showed that other negative emotions (other than anxiety) appear even more frequently: anger (50.7%), annoyance at the inconvenience of the disease (50.0%), irritation (47%), and shame (46.7%) [34].

Taking into consideration the presence of depressive symptoms, we found that patients with no depression had the highest satisfaction with the patient–doctor relationship. The assessment of the actual patient–doctor relationship was the lowest among patients with severe depression. This is in line with the findings by Drenkard et al. [35] An IPC-29 instrument, together with the PHQ-9 scale, was used amid patients with lupus erythematosus. They found significant linear trends of poorer scores for all communication scales across more severe disease activity and depression symptoms and lower scores for all interpersonal style scales across more severe lupus erythematosus activity [35]. Moreover, we have established that not only are the level of anxiety and depressive symptoms important, but somatic symptoms, insomnia, and social dysfunction (in the sense of being busy, managing tasks, and day-to-day activities) also have great importance in the assessment of the patient–doctor relationship. The greater those symptoms, the lower the satisfaction as well as the actual patient–doctor relationship assessment. 

Physician–patient interactions can be measured by other scales such as the Interpersonal Process of Care (IPC-29) scale, as used by the Drenkard et al. study cited above [35]. However, IPC-29 does not take into consideration the ideal, only the current, relation/communication with the doctor. The advantages of the Patient Expectation Test [22] applied in our study are the possibilities to additionally evaluate the ideal relationships and satisfaction with the patient–doctor interaction. Similarities among those tests are the assessments of whether the physician explained and provided information regarding therapeutic options [35].

A study by Renzi et al. [33] conducted on a cohort of 396 dermatological outpatients (whose most frequent diagnoses were dermatitis, acne, and naevi) showed that 60% of patients were satisfied with their dermatologists. In this paper, satisfaction was determined by the doctor’s ability to explain and empathize, as well as the patient’s age, with the older patients being more satisfied. Despite the similar mean age of our study groups (ours and Renzi et al. [33]), in our cohort, age as well as sex had no significant influence on the satisfaction with the patient–doctor relationship. Contrary to our study, where the assessment of the satisfaction with the patient–doctor relationship was not dependent on the severity of the disease, the paper by Renzi et al. [33] showed that satisfaction was higher among individuals with more severe disease. We suspect that the major difference could be due to the smaller cohort of patients in our study or the difference in the type of measurement tools utilized. However, the most important difference is the heterogeneity of the participants of the Renzi et al. [33] study.

Our team is mindful of the limitations of our study. This study was only performed in two different regions of Poland. Thus, the results should not be generalized. The screening of psychological symptoms was not confirmed by a detailed psychiatric examination. Additionally, a team of dermatologists took care of the patients with HS. It is of note that the assessment of SWL could be influenced by other parameters that have not been examined, such as personality traits, life experiences, trauma, and others, which is an interesting area for research in the future. There are additional factors that should be taken into consideration, such as weaknesses of the subjective assessment based on self-assessment, the respondent’s tiredness, misunderstanding of questions, and negative feelings of patients, such as malice. These modalities can create respondent bias. However, despite the anonymous questionnaires, patients can also offer a different respondent bias, where they offer positive responses, making the “satisfaction with the patient–doctor relationship” a clearly subjective outcome variable. On the other hand, we can defend the subjective assessments and patients’ self-reports if our main aim is patient-centered medicine and a holistic approach to the treatment. 

## 5. Conclusions

Our findings highlight the importance and complexity of the patient–doctor relationship among HS patients. Paying special attention to this aspect, usage of the Patient Expectation Test in daily clinical practice can greatly improve the patient–doctor partnership and, ultimately, the general quality of care and compliance. Factors unrelated to the particular disease state were of greater importance. They include mental health issues, which we proved should always be considered in everyday patient care. We believe that our study brings a new perspective on this important topic among HS patients. 

## Figures and Tables

**Table 1:** Demographic and clinical characteristics of hidradenitis suppurativa patients.

Characteristics	Overall N = 105	Females N = 56 (53%)	Males N = 49 (47%)	*p*
Age	38.32, (13.30)	37.64, (14.01)	39.10, (12.52)	0.51
Duration of the disease	9.53, (8.17)	10.89, (8.14)	7.98, (8.00)	0.016
Number of hospitalizations	1.68, (2.66)	1.89, (3.27)	1.43, (1.68)	0.54
Hurley stages				0.30
I	26 (24.8%)	14 (25.0%)	12 (24.5%)	
II	69 (65.7%)	39 (69.6%)	30 (61.2%)	
III	10 (9.5%)	3 (5.4%)	7 (14.3%)	
IHS4 severity stage				0.87
Mild	25 (23.8%)	14 (25.0%)	11 (22.4%)	
Moderate	38 (36.2%)	19 (33.9%)	19 (38.8%)	
Severe	42 (40.0%)	23 (41.1%)	19 (38.8%)	

N—number of patients; SD—standard deviation; IHS4—International Hidradenitis Suppurativa Score System.

**Table 2:** Patient–doctor relationship among individuals with hidradenitis suppurativa.

The Patient Expectation Test	Females N = 56 (53%)	Males N = 49 (47%)	*p*
Patient–doctor relationship			
Ideal	27.96, (3.47)	27.71, (3.59)	0.6
Actual	24.96, (5.44)	26.44, (5.03)	0.15
Satisfaction	3.00, (5.94)	1.27, (4.87)	0.084

N—number of patients; IHS4—International Hidradenitis Suppurativa Severity Score; Ideal—ideal patient–doctor relationship; Actual—actual patient–doctor relationship; Satisfaction—satisfaction with the patient–doctor relationship; *p*—*p*-value.

**Table 3:** Patient–doctor relationship and satisfaction with life among individuals with hidradenitis suppurativa.

	SWLS			
Characteristic	Low N = 41 (39%)	Intermediate N = 40 (38%)	High N = 24 (23%)	*p*
Ideal	28.02, (3.29)	27.90, (3.62)	27.25, (3.84)	0.63
Actual	23.24, (6.21)	27.10, (3.68)	27.38, (4.22)	0.006
Satisfaction	4.78, (6.39)	0.80, (4.00)	−0.13, (4.33)	0.004

N—number of patients; SWLS—Satisfaction with Life Scale; *p*—*p*-value; Ideal—ideal patient–doctor relationship; Actual—actual patient–doctor relationship; Satisfaction—satisfaction with the patient–doctor relationship.

**Table 4:** Patient–doctor relationship and depressive symptoms among individuals with hidradenitis suppurativa.

	PHQ-9					
Characteristic	None N = 62 (59%)	Mild N = 17 (16%)	Moderate N = 19 (18%)	Moderately Severe N = 4 (3.8%)	Severe N = 3 (2.9%)	*p*
Ideal	27.90, (3.64)	27.59, (3.26)	27.05, (3.46)	29.50, (3.70)	29.33, (3.79)	0.6
Actual	27.27, (3.85)	25.18, (5.68)	23.63, (4.78)	21.75, (9.00)	13.00, (2.65)	0.001
Satisfaction	0.63, (3.85)	2.41, (5.46)	3.42, (5.81)	7.75, (9.18)	16.33, (3.21)	0.002

N—number of patients; PHQ-9—Patient Health Questionnaire-9; *p*—*p*-value; Ideal—ideal patient–doctor relationship; Actual—actual patient–doctor relationship; Satisfaction—satisfaction with the patient–doctor relationship.

**Table 5:** Patient–doctor relationship and anxiety symptoms among individuals with hidradenitis suppurativa.

	GAD-7				
Characteristic	None N = 62 (59%)	Mild N = 28 (27%)	Moderate N = 10 (9.5%)	Severe N = 5 (4.8%)	*p*
Ideal	27.79, (3.63)	27.11, (3.44)	28.20, (3.22)	31.00, (1.73)	0.11
Actual	27.05, (4.06)	24.68, (5.89)	24.00, (5.94)	17.20, (4.55)	0.003
Satisfaction	0.74, (4.07)	2.43, (5.47)	4.20, (6.29)	13.80, (6.06)	0.001

Ideal—ideal patient–doctor relationship; Actual—actual patient–doctor relationship; Satisfaction—satisfaction with the patient–doctor relationship; GAD-7—General Anxiety Disorder Scale-7; N—number of patients; *p*—*p*-value.

**Table 6:** Multiple regression analysis for the actual patient–doctor relationship and the satisfaction with the patient–doctor relationship of individuals with hidradenitis suppurativa.

Characteristic	Actual Patient–Doctor Relationship			Satisfaction with Patient–Doctor Relationship		
	Beta	95% CI	*p*	Beta	95% CI	*p*
Hurley:						
grade I	—	—		—	—	
grades II + III	2.9	0.71, 5.2	0.010	−3.4	−5.7, −1.2	0.003
GHQ-28	−0.20	−0.32, −0.08	0.001	0.20	0.08, 0.33	0.001
GAD-7:						
mild + moderate	—	—		—	—	
none	−1.2	−3.7, 1.4	0.365	1.2	−1.3, 3.8	0.344
severe	−4.9	−9.4, −0.37	0.034	6.1	2.8, 12	0.002

Beta—regression coefficient; 95% CI—Confidence Interval; *p*—*p*-value; GHQ-28—General Health Questionnaire-28; GAD-7—General Anxiety Disorder divided into 3 levels: mild + moderate, none, and severe.

## Data Availability

Data supporting the reported results can be obtained on request; e-mail: julia.rymaszewska@student.umw.edu.pl.
